# Single-cell and WGCNA uncover a prognostic model and potential oncogenes in colorectal cancer

**DOI:** 10.1186/s12575-022-00175-x

**Published:** 2022-09-19

**Authors:** Ziyang Di, Sicheng Zhou, Gaoran Xu, Lian Ren, Chengxin Li, Zheyu Ding, Kaixin Huang, Leilei Liang, Yihang Yuan

**Affiliations:** 1 Zhongnan Hospital of Wuhan University, Wuhan, China; 2 National Cancer Center/National Clinical Research Center for Cancer/Cancer Hospital, Chinese Academy of Medical Sciences and Peking Union Medical College, Beijing, China; 3 Department of General Surgery, Tongren Hospital, Shanghai Jiao Tong University School of Medicine, 1111 XianXia Road, Shanghai, 200336 China

**Keywords:** Colorectal cancer, Single cell, WGCNA, Prognostic model

## Abstract

**Background:**

Colorectal cancer (CRC) is one of the leading causes of cancer-related death worldwide. Single-cell transcriptome sequencing (scRNA-seq) can provide accurate gene expression data for individual cells. In this study, a new prognostic model was constructed by scRNA-seq and bulk transcriptome sequencing (bulk RNA-seq) data of CRC samples to develop a new understanding of CRC.

**Methods:**

CRC scRNA-seq data were downloaded from the GSE161277 database, and CRC bulk RNA-seq data were downloaded from the TCGA and GSE17537 databases. The cells were clustered by the FindNeighbors and FindClusters functions in scRNA-seq data. CIBERSORTx was applied to detect the abundance of cell clusters in the bulk RNA-seq expression matrix. WGCNA was performed with the expression profiles to construct the gene coexpression networks of TCGA-CRC. Next, we used a tenfold cross test to construct the model and a nomogram to assess the independence of the model for clinical application. Finally, we examined the expression of the unreported model genes by qPCR and immunohistochemistry. A clone formation assay and orthotopic colorectal tumour model were applied to detect the regulatory roles of unreported model genes.

**Results:**

A total of 43,851 cells were included after quality control, and 20 cell clusters were classified by the FindCluster () function. We found that the abundances of C1, C2, C4, C5, C15, C16 and C19 were high and the abundances of C7, C10, C11, C13, C14 and C17 were low in CRC tumour tissues. Meanwhile, the results of survival analysis showed that high abundances of C4, C11 and C13 and low abundances of C5 and C14 were associated with better survival. The WGCNA results showed that the red module was most related to the tumour and the C14 cluster, which contains 615 genes. Lasso Cox regression analysis revealed 8 genes (PBXIP1, MPMZ, SCARA3, INA, ILK, MPP2, L1CAM and FLNA), which were chosen to construct a risk model. In the model, the risk score features had the greatest impact on survival prediction, indicating that the 8-gene risk model can better predict prognosis. qPCR and immunohistochemistry analysis showed that the expression levels of MPZ, SCARA3, MPP2 and PBXIP1 were high in CRC tissues. The functional experiment results indicated that MPZ, SCARA3, MPP2 and PBXIP1 could promote the colony formation ability of CRC cells in vitro and tumorigenicity in vivo*.*

**Conclusions:**

We constructed a risk model to predict the prognosis of CRC patients based on scRNA-seq and bulk RNA-seq data, which could be used for clinical application. We also identified 4 previously unreported model genes (MPZ, SCARA3, MPP2 and PBXIP1) as novel oncogenes in CRC. These results suggest that this model could potentially be used to evaluate the prognostic risk and provide potential therapeutic targets for CRC patients.

**Supplementary Information:**

The online version contains supplementary material available at 10.1186/s12575-022-00175-x.

## Introduction

Colorectal cancer (CRC) has the third highest incidence and the second highest mortality worldwide [1]. The five-year survival rate is less than 15%, which is linked to the spread of the cancer and the failure of early diagnosis [2]. Alterations at the genetic and epigenetic levels have been recognized as major players in CRC initiation and development [3]. Therefore, a better understanding of CRC molecular mechanisms is urgently needed to develop CRC diagnosis and better treatment strategies.

In recent decades, bulk transcriptome sequencing (bulkRNA-seq) has been a powerful technique to identify new molecular biomarkers and improve our understanding of tumour development. For instance, Xu M et al. revealed the functions of SATB2-AS1 in CRC progression, suggesting new biomarkers and therapeutic targets in CRC [4]. In addition, our previous study identified a 4-gene prognostic model predicting survival in CRC [5]. However, traditional RNA-seq is mainly concentrated on the “average” expression of all cells, an approach that cannot detect the molecular complexity and diversity of tumour cells in a sample. Currently, single-cell RNA-sequencing (scRNA-seq) technology elucidates the molecular distinction of all cell type compositions and enables cell population profiling of tumours at single-cell resolution. scRNA-seq provides deeper insights into transcriptome expression profiles at a single-cell resolution and is applied to develop personalized therapeutic strategies that are potentially useful in cancer diagnosis and therapy resistance during cancer progression [6, 7]. Using scRNA-seq, Siel Olbrecht et al. identified marker genes specific for stromal cell phenotypes predicting overall survival in high-grade serous tubo-ovarian cancer patients [8]. Additionally, Katzenelenbogen Y et al. revealed an immunosuppressive role of TREM2 by coupling scRNA-Seq and intracellular protein activity in cancer [9]. Therefore, to comprehensively identify the predictive biomarkers and novel molecular targets of gene therapy for CRC, utilizing bulkRNA-seq and scRNA-seq analysis could precisely stratify patients and recognize patients.

In this study, we aimed to construct a prognostic model for patients with CRC by integrating scRNA-seq and bulk RNA-seq data. The capability of this model in predicting the prognosis of CRC was validated. Furthermore, the results of qPCR and immunohistochemistry staining assays revealed that MPZ, SCARA3, MPP2 and PBXIP1 were upregulated in CRC tissues. Functional studies showed that the over-expression of MPZ, SCARA3, MPP2 and PBXIP1 could promote the colony formation abilities of CRC cells in vitro and in vivo. We believe our findings will provide a potential prognostic model and therapeutic targets for CRC.

## Methods

### Data download

CRC scRNA-seq data were downloaded from the GEO Database, accession number GSE161277, which included 13 samples. CRC bulk RNA-seq data were downloaded from TCGA (431 tumour and 41 normal samples) and GSE17537 (55 tumour samples) databases.

### scRNA-seq data processing and cell type identification

First, the scRNA-seq data were filtered by setting each gene to be expressed in at least 3 cells, and each cell expressed at least 250 genes. Second, we calculated the proportions of mitochondria and rRNA through the PercentageFeatureSet function. We ensured that the genes expressed in each cell were between 100 and 7500, the mitochondrial content was less than 35%, and the Unique Molecular Identifiers (UMI) of each cell was more than 1000; as a result, 43,851 cells were obtained. Fig. S1A-D shows the quality control charts before and after filtration.

Then, we normalized the data of the 13 samples separately by log-normalization. The FindVariableFeatures function was used to find hypervariable genes (variable features were identified based on variance-stabilizing transformation (“vst”)), and then, the batch effect of the samples was removed using the FindIntegrationAnchors function of the canonical correlation analysis (CCA) method. Furthermore, we used the IntegrateData function to integrate the data, the ScaleData function to scale all genes, and principal component analysis (PCA) dimensionality reduction to find anchor points (Fig. S1E). We selected dim = 30, and the cells were clustered by the FindNeighbors and FindClusters function (Resolution = 0.4), and 20 clusters were obtained. Furthermore, we downloaded the marker genes of human cells from the CellMarker website (http://biocc.hrbmu.edu.cn/CellMarker/) [10] and selected the list of tissue and cell marker genes related to colon, internal crypt, gut, peripheral blood and blood. The FindCluster () function was used to cluster cells.

### Abundance analysis of clusters in bulk RNA-seq samples

To assess the correlation between cell types obtained by scRNA-seq and bulk RNA-seq, CIBERSORTx (version R) was applied to detect the abundance of cell clusters in the bulk RNA-seq expression matrix, which is expressed as log2(TPM + 1) normalized.

### WGCNA analysis

WGCNA with the expression profile using the R package “WGCNA” was used to construct the gene coexpression networks of TCGA-CRC (431 tumour and 41 normal samples). The network construction process mainly includes the following steps: 1. Define the similarity matrix. 2. Select the weight coefficient β = 12 to convert the similarity matrix into an adjacency matrix. 3. Transform the adjacency matrix into a topological overlap matrix (TOM). 4. Layer the dissTOM based on Tom Cluster to obtain a hierarchical clustering tree. 5. A dynamic tree-cut method was used to identify modules from the hierarchical clustering tree. 6. We calculated the module eigengenes (MEs) for each module, where MEs represent the overall expression level of the module. The Pearson correlation coefficient between the MEs of each module was calculated, and the 1-Pearson correlation coefficient was defined as the average distance between the MEs of each module. The MEs of all modules were clustered using the average linkage hierarchical clustering method, and the minimum value (genome) was set to 100. The modules with high similarity were combined to obtain a coexpression network.

### Cell trajectory and cell communication analysis

To characterize the underlying processes of functional changes and identify potential lineage differentiation between different clusters, Monocle2 was used to perform a pseudotemporal analysis of cellular evolution. The Monocle2 algorithm was used to calculate the pseudotime, and the resulting pseudotime was scaled from 0 to 1. Then, the hub genes in each cluster were identified by the “differentialGeneTest”. The expression profile was reduced to 2 dimensions (max_components = 2) by the “reduceDimension” function and the DDRTree method. Then, the “orderCells” function was used to sort the cells and assign “pseudotime” values.

To study the communication interaction between cells and identify the mechanism of communication molecules at single-cell resolution, the R package “CellChat” was used to perform cell–cell communication correlation analysis.

### Construction of the prognostic risk model

We calculated the risk score for each patient using the following formula:

$$\mathrm{RiskScore}=\sum \mathrm{betai}\times \mathrm{Expi}$$

i refers to the expression level of the gene, and beta is the coefficient of the receptor–ligand pair of multivariate Cox regression. Based on the threshold (median value), patients were divided into high- and low-risk groups, survival curves were drawn using the Kaplan–Meier method for prognostic analysis, and the log-rank test was used to determine the significance of differences.

### Tissue specimens

Fresh CRC tissues and adjacent normal tissues were collected from the Department of Gastrointestinal Surgery, Zhongnan Hospital of Wuhan University. No patients received treatment before surgery, and all patients signed informed consent forms provided by Zhongnan Hospital. The primary tumour area and morphologically normal surgical margin tissue were immediately isolated from each patient by an experienced pathologist and stored in liquid nitrogen until use. The study was approved by the Zhongnan Hospital of Wuhan University (2,017,047).

### Cell culture

The human CRC cell lines SW480 and SW620 were purchased from the ATCC Cell Bank of the United States. SW480 and SW620 cells were grown in Leibovitz’s L-15 medium (Sigma, Beijing, China) supplemented with 10% foetal bovine serum (FBS) (Gibco, Grand Island, NY, USA), 2 mmol/l glutamine, 100 U/ml penicillin, and 100 μg/ml streptomycin (Thermo Scientific, Waltham, MA, USA) in a 37 °C incubator without CO_2_.

### Transfection and lentiviral transduction

Lentiviral expression vectors (pLKO.1-NC-GFP, pLKO.1-MPZ-GFP, pLKO.1-SCARA3-GFP, pLKO.1-MPP2-GFP or pLKO.1-PBXIP1-GFP) and packaging plasmids (psPAX2 and pMD2. G) were used to construct MPZ-, SCARA3-, MPP2- or PBXIP1-interfering SW620 cell lines. Lentiviral expression vectors (pLVX-IRES-Neo-3xFlag, pLVX-MPZ-IRES-Neo-3xFlag, pLVX-SCARA3-IRES-Neo-3xFlag, pLVX-MPP2-IRES-Neo-3xFlag or pLVX-PBXIP1-IRES-Neo-3xFlag) and packaging plasmids (psPAX2 and pMD2. G) were used for overexpression of MPZ, SCARA3, MPP2 or PBXIP1 in the CRC cell line SW480. Cells were transfected with interfering expression or overexpression plasmids using Lipofectamine 2000 (12,566,014, Invitrogen, Shanghai, China) following the manufacturer’s protocols. To generate the lentivirus, 293FT cells were cotransfected with psPAX2, pMD2G, pLKO.1-MPZ-GFP, pLKO.1-SCARA3-GFP, pLKO.1-MPP2-GFP or pLKO.1-PBXIP1-GFP to interfere with expression. 293FT cells were cotransfected with pLVX-MPZ-IRES-Neo-3xFlag, pLVX-SCARA3-IRES-Neo-3xFlag, pLVX-MPP2-IRES-Neo-3xFlag or pLVX-PBXIP1-IRES-Neo-3xFlag for overexpression. Forty-eight hours after transfection, the lentiviral supernatants were collected and filtered through a 0.45-μm filter. The lentiviruses were added to medium containing 8 µg/ml polybrene (Sigma, St. Louis, MO, USA) and transduced into CRC cells according to the manufacturer’s instructions. Stable cells were selected for at least 1 week using PURO (P8230-25, Solarbio, Beijing, China) or G418 (10,131,035, Invitrogen, Shanghai, China).

### Total RNA extraction and quantitative real‑time PCR

Total RNA extraction was performed using RNA-easy Isolation Reagent (No. RC112-01, Vazyme, China). Then, quantitative real-time PCR (qRT‒PCR) was performed using the HiScript III 1st Strand cDNA Synthesis Kit (No. R312-01, Vazyme, China) and ChamQTM Universal SYBR® qPCR Master Mix (No. Q712-02, Vazyme, China) according to the manufacturer’s instructions. The primer sequences were as follows: MPZ Forward Sequence 5’-3’: CTATCCTGGCTGTGCTGCTCTT and Reverse Sequence 5’-3’: ACTCACTGGACCAGAAGGAGCA; SCARA3 Forward Sequence 5’-3’: CTCCGAAGACATCTCCTTGACC and Reverse Sequence 5’-3’:CCAGCTTCATGGCAGAAAGAGC; MPP2 Forward Sequence 5’-3’:GGCACACGTATTGACTCCATCC and Reverse Sequence 5’-3’: GCCTCGATGAACACCACGTAAG; PBXIP1 Forward Sequence 5’-3’: ACGCTCTTCCAGACTGAAAGCCACTGC and Reverse Sequence 5’-3’: TCCCTGGACTACTGTGTCTCCT; GAPDH Forward Sequence 5’-3’: GTCTCCTCTGACTTCAACAGCG and Reverse Sequence 5’-3’: ACCACCCTGTTGCTGTAGCCA. GAPDH served as an internal control.

### Immunohistochemistry

An immunohistochemistry (IHC) staining SP kit (No. SP-9000, ZSGB-BIO, Beijing, China) was used for IHC, which was performed as previously described [5]. Anti-MPZ (ab183868, Abcam, Shanghai, China) (1:200), anti-MPP2 (1:200) (ab231634, Abcam, Shanghai, China) and anti-PBXIP1 (ab84752, Abcam, Shanghai, China) (1:200) were purchased from Abcam. Anti-SCARA3 (sc-365649, SANTA CRUZ, Shanghai, China) (1:200) was purchased from SANTA CRUZ. The magnification of the IHC images was 20 × .

### Colony formation assay

Cells were seeded into 6-well plates at 1 × 10^3^ cells per plate. The cells were mixed and then cultured for 10 days with culture medium containing L-15 with 10% FBS. The following criterion was considered for evaluating the results: clusters of ≥ 30 cells were counted as a colony.

### Xenograft model tumour assay

Antitumor therapy in an orthotopic colorectal tumour model. The orthotopic CRC model was developed in female BALB/c nude mice as described previously [9]. Female BALB/c nude mice (4–5 weeks old) were anaesthetized by an intraperitoneal injection of ketamine-xylazine solution. The abdomen was sterilized with alcohol swabs. A median incision was then made through the lower ventral abdomen, and the caecum was exteriorized. A suspension of 2 × 10^6^ SW620-luc cells/SW480-luc cells in 50 µL serum-free DMEM containing 10 µg µL-1 Matrigel was injected into the caecal wall using a 30 G needle (Hamilton Company, Reno, NV). To prevent leakage, a cotton swab was carefully held over the injection site for 1 min. The caecum was then returned to the peritoneal cavity, and the peritoneum and skin were closed with 5–0 suture. Tumour formation and growth were monitored using a Xenogen IVIS 200 imaging system (Caliper Life Sciences, MA). At the end of the experiment (after 4 weeks), the mice were euthanized by an intraperitoneal injection of 100 mg/kg pentobarbital sodium (Sigma, St. Louis, MO, USA).

### Statistical analysis

All statistical analyses were performed using R software 3.5.3 and GraphPad Prism v. 8.01 (GraphPad Software, La Jolla, CA, USA). Student’s t test was used to compare values between the test and control groups, and *P* < 0.05 was considered significant.

## Results

### Dimensionality reduction clustering of CRC single-cell data

**Fig. 1:**
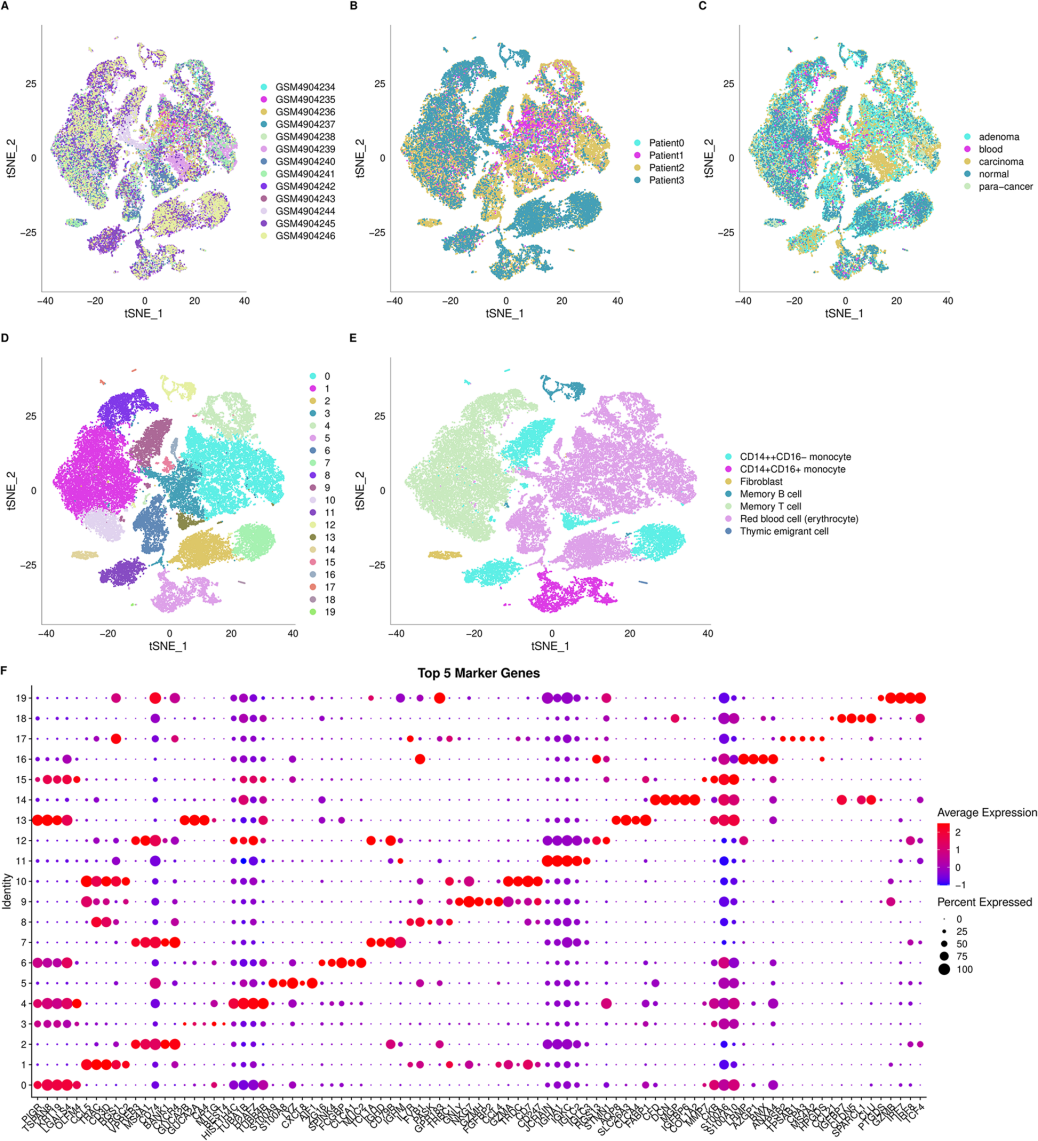
The characteristics of different cell groups of CRC. **A** The tsne map of 13 mixed samples. **B** The tsne diagram of four patients and **C** tumor types are also mixed together. **D** The FindCluster () function was used to obtain 20 clusters. **E** Twenty clusters annotated to 7-cell types by ssGSEA. **F** The expression of the top five marker genes with the most prominence in each subgroup

To study the characteristics of different cell groups of CRC, we collected the scRNA-seq profiles of 43,851 cells. As shown in the tsne map of 13 samples (Fig. 1A), the samples were mixed together. The tsne diagram of four patients (Fig. 1B) and tumour types (Fig. 1C) were also mixed together. Furthermore, the FindCluster () function was used to cluster cells and obtain 20 clusters (Fig. 1D). Next, we annotated the 20 clusters by ssGSEA (Table 1) to 7 cell types (Fig. 1E) (CD14 +  + CD16- monocytes, CD14 + CD16 + monocytes, fibroblasts, memory B cells, memory T cells, red blood cells (erythrocytes) and thymic embryonic cells). The logfc = 0.35, Minpct = 0.15 and padj < 0.05 of FindAllMarkers were set to screen marker genes of the 20 clusters. The expression levels of the top five marker genes with the most prominence in each subgroup are shown in Fig. 1F, and the results of the marker genes are shown in Supplementary Table S1.

**Table 1:** Clusters annotate

seurat_clusters	cell_type
C0	Red blood cell (erythrocyte)
C1	Memory T cell
C10	Memory T cell
C11	CD14 + + CD16- monocyte
C12	Memory B cell
C13	CD14 + + CD16- monocyte
C14	Fibroblast
C15	Red blood cell (erythrocyte)
C16	Red blood cell (erythrocyte)
C17	CD14 + + CD16- monocyte
C18	Thymic emigrant cell
C19	CD14 + CD16 + monocyte
C2	Red blood cell (erythrocyte)
C3	Red blood cell (erythrocyte)
C4	Red blood cell (erythrocyte)
C5	CD14 + CD16 + monocyte
C6	Red blood cell (erythrocyte)
C7	CD14 + + CD16- monocyte
C8	Memory T cell
C9	CD14 + + CD16- monocyte

**Fig. 2:**
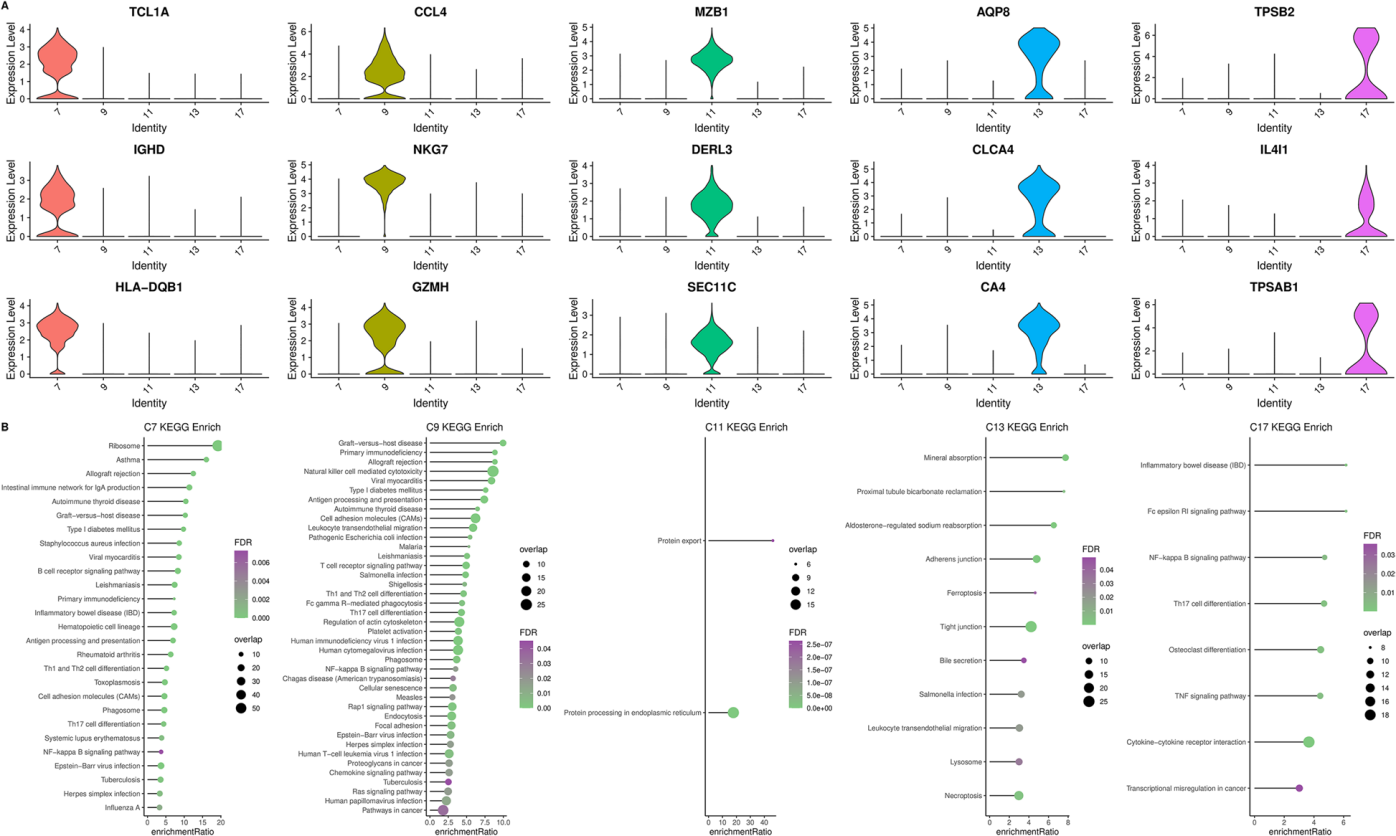
The cell types of the cluster marker genes. **A** The first three marker genes to draw a violin diagram and **B** We screened the key pathways by FDR < 0.05 in these 5 clusters (C7, C9, C11, C13 and C17)

To better interpret and distinguish these 20 clusters, we analysed the cell types of the cluster marker genes. The results show the following: 1. Five clusters (C7, C9, C11, C13 and C17) were annotated as CD14 +  + CD16- monocyte cells. We selected the first three marker genes to draw a violin diagram (Fig. 2A). At the same time, we performed KEGG annotation through the WebGestaltR package and screened the key pathways by FDR < 0.05 in these 5 clusters (Fig. 2B). 2. Two clusters (C5 and C19) were annotated as CD14 + CD16 + monocyte cells (Fig. S2 A-B). 3. Three clusters (C1, C8 and C10) were annotated as memory T cells (Fig. S2 C-D). 4. Seven clusters (C0, C2, C3, C4, C6, C15 and C16) were annotated as red blood cells (erythrocytes) (Fig. S3 A-B). 5. C14 were annotated as fibroblast cells, C12 were annotated as memory B cells and C18 were annotated as thymic emigrant cells (Fig. S4). The results of the 20-cluster enrichment analysis are shown in Supplementary Table S2.

### Prediction and analysis of cellular cluster abundance based on TCGA databases

**Fig. 3:**
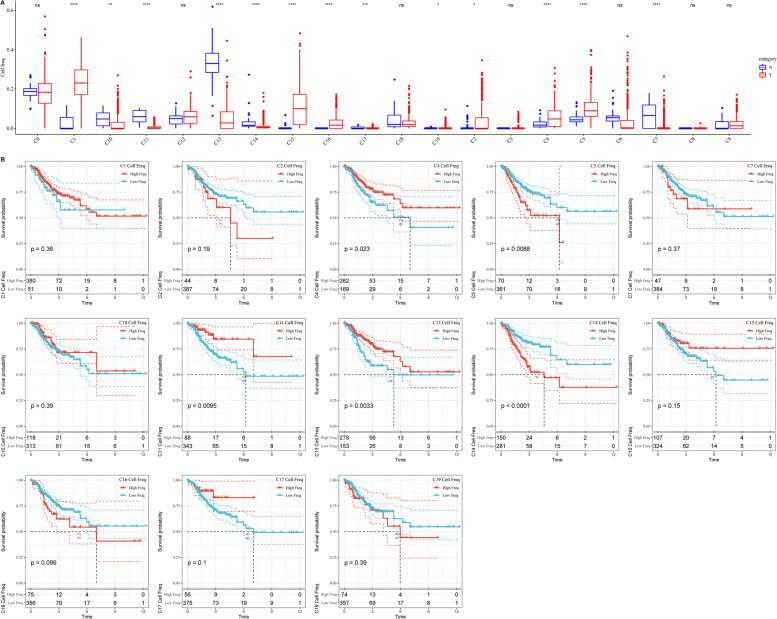
The abundance of 20 clusters in TCGA-CRC databases. **A** The abundance of C1, C2, C4, C5, C15, C16 and C19 was high, and the abundance of C7, C10, C11, C13, C14 and C17 was low in CRC tumor tissues. **B** The results of survival analysis showed that high abundances of C4, C11 and C13 and low abundances of C5 and C14 were associated with better survival

CIBERSORT was used to analyse the abundances of the 20 clusters in TCGA-CRC databases. We found that the abundances of 13 clusters were significantly different between CRC tumour and normal tissues. The abundances of C1, C2, C4, C5, C15, C16 and C19 were high and the abundances of C7, C10, C11, C13, C14 and C17 were low in CRC tumour tissues (Fig. 3A). Meanwhile, the results of survival analysis showed that high abundances of C4, C11 and C13 and low abundances of C5 and C14 were associated with better survival (Fig. 3B). At the same time, we found a significant correlation between C14 and patient prognosis.

**Fig. 4:**
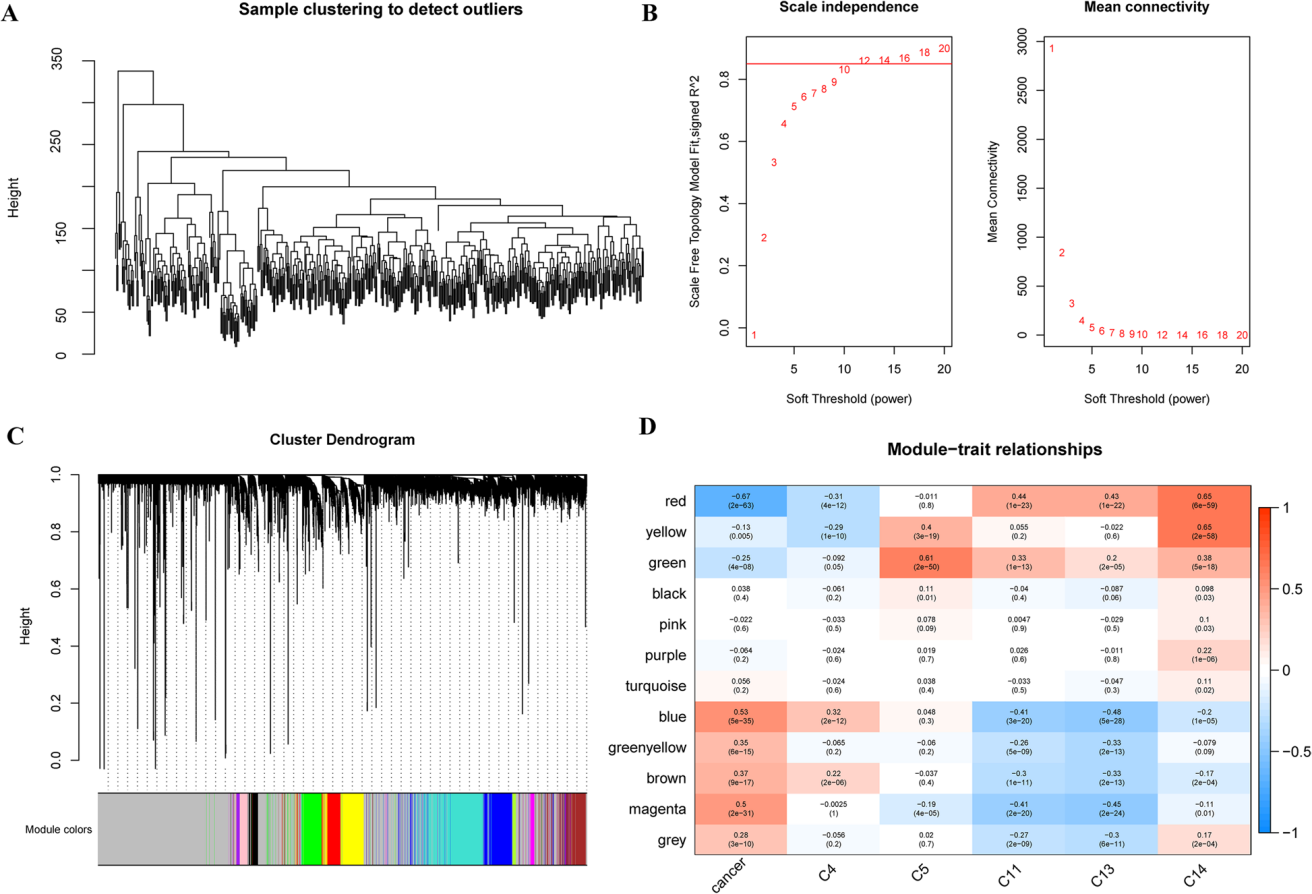
We applied WGCNA to construct the gene coexpression networks of CRC patients. **A** A weighted coexpression network. **B** β = 12 to ensure that the network is scale-free. **C** A total of 12 modules were obtained. **D** The red module is most related to the tumor and C14 cluster

We applied WGCNA with the expression profile using the R package “WGCNA” to construct the gene coexpression networks of CRC patients. Pearson’s correlations were performed for all pairwise genes, and WGCNA was used to build a weighted coexpression network (Fig. 4A). We next calculated 5 as the optimal soft threshold for the adjacency computation. In the present study, the coexpression network conformed to the scale-free network, and we chose β = 12 to ensure that the network was scale-free (Fig. 4B).

Next, the expression matrix was transformed into an adjacency matrix, and then the adjacency matrix was transformed into a topology matrix. Based on TOM, we used the average-linkage clustering method to cluster genes and set 100 as the minimum number of genes in each module according to the standard of the hybrid dynamic clipped tree. We performed cluster analysis on the modules and merged the modules with closer distances into a new module (set height = 0.25, deepSplit = 3, minModuleSize = 100), and a total of 12 modules were obtained (Fig. 4C). In cluster analysis, the grey module represented a gene set that could not be aggregated into other modules. We further analysed the correlation between each module and the abundances of C4, C5, C11, C13 and C14 (Fig. 4D). The results showed that the red module was most related to the tumour and the C14 cluster. The red module contains 615 genes and is shown in Supplementary Table S3.

**Fig. 5:**
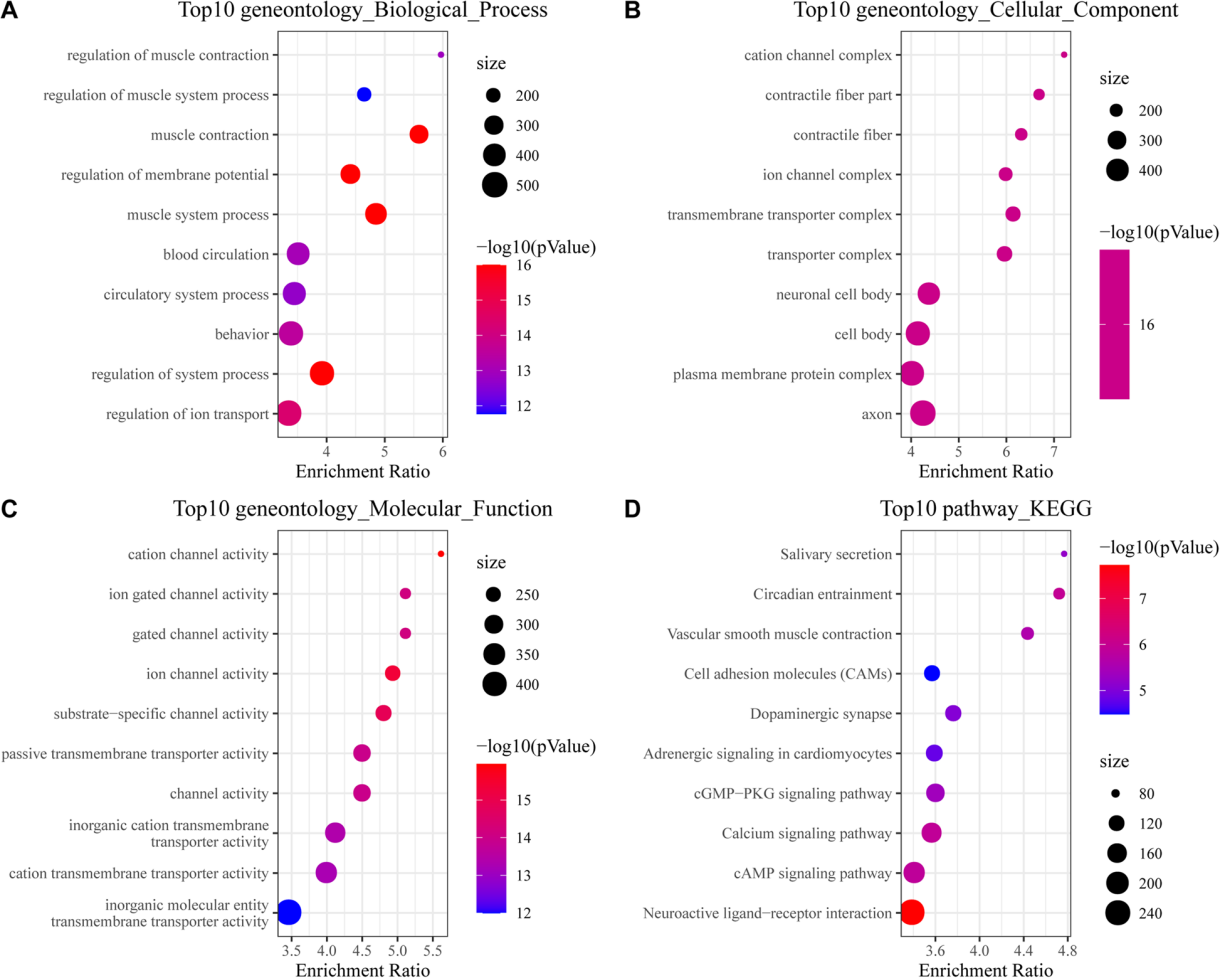
GO functional enrichment and KEGG pathway analysis in the red module. **A** The top 10 significant differences in Biological Process. **B** The top 10 significant differences in cellular components. **C** The top 10 significant differences in Molecular Function. **D** The results of the first 10 pathways

We chose the red module for more detailed analysis, and the R software package “WebGestaltR” was used for GO functional enrichment and KEGG pathway analysis (Supplementary Table S4). For the GO functional enrichment: 1. A total of 237 gene ontologies were annotated with significant differences in biological process (BP) (FDR < 0.05), and the top 10 are shown in Fig. 5A. 2. A total of 122 genes were annotated with significant differences in cellular components (CCs) (FDR < 0.05), and the top 10 are shown in Fig. 5B. 3. Fifty-nine gene ontologies were annotated with significant differences in molecular function (MF) (FDR < 0.05), and the top 10 are shown in Fig. 5C. For KEGG pathway enrichment of marker genes, a total of 28 pathways were significantly annotated (FDR < 0.05), and the results of the first 10 pathways are shown in Fig. 5D. These annotation results showed that these genes were closely related to tumorigenesis.

### Cell communication analysis of key clusters

**Fig. 6:**
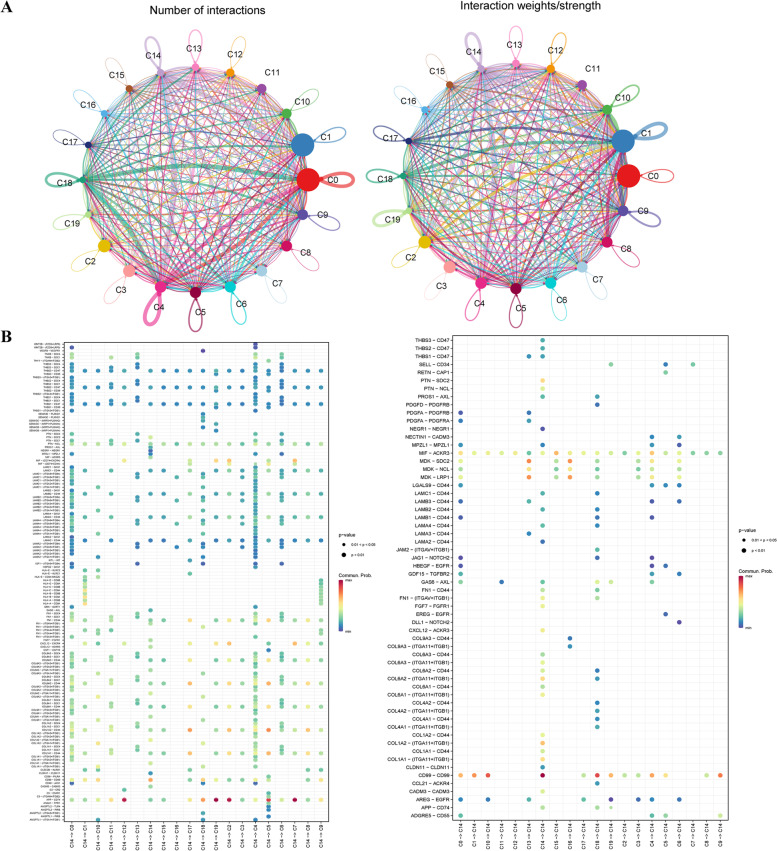
The cellular communication between C14 and other clusters. **A** Among the 20 clusters, there were high cell-to-cell correlations in terms of the number and intensity of ligand–receptor interactions. **B** The effects were also found in C14 clusters by others, such as C13 and C16 clusters affecting C14 via MDK-SDC2

In multicellular organisms, the basic process of cell life activities depends on cell–cell interactions, and the communication between cells is mostly mediated by multisubunit protein complexes. Based on the above analysis results, we found that the C14 cluster plays an important role in the tumorigenesis of CRC and analysed the cellular communication between C14 and other clusters. We used CellCharts to analyse the cell communication among the 20 cell clusters, and the results are shown in Supplementary Table S5. Among the 20 clusters, there were high cell-to-cell correlations in terms of the number and intensity of ligand–receptor interactions (Fig. 6A). By extracting the ligand–receptor information of each cluster, we found that C14 affects other clusters through ligand receptors; for example, C14 affects the C1 cluster through HLA-C-CD8A and clusters C2, C7, C12, and C19 by APP-CD74. The effects were also found in C14 clusters by others, such as C13 and C16 clusters affecting C14 via MDK-SDC2 (Fig. 6B).

### Construction and evaluation of a prognostic risk model

**Fig. 7:**
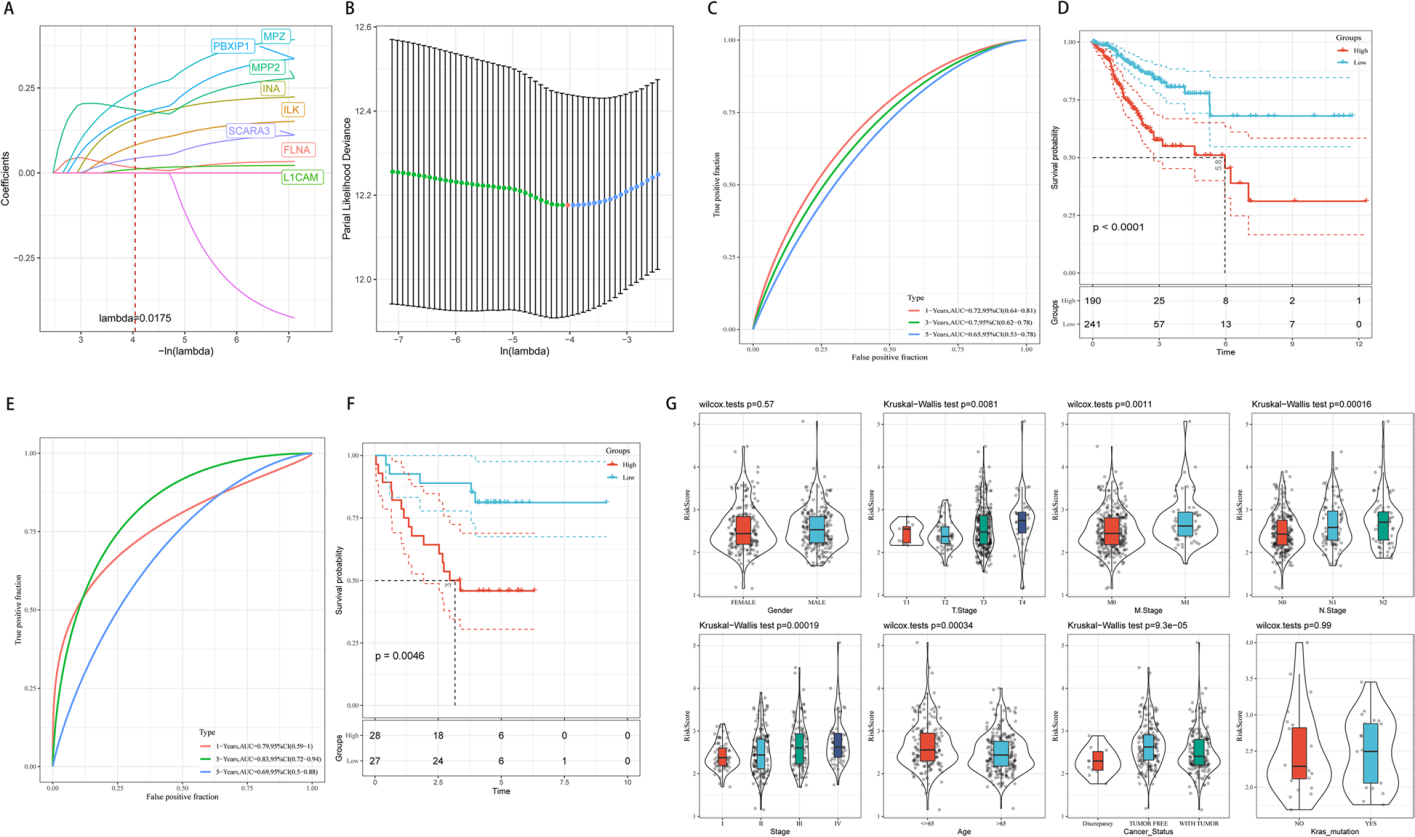
Construction and evaluation of a prognostic risk model. **A-B** The model was optimal when lambda = 0.0175, and 8 genes were chosen to construct a risk model. **C** The AUCs of the risk model for predicting 1-, 3- and 5-year survival were 0.72, 0.70 and 0.65, respectively, and **D** patients with a high risk score presented significantly worse OS than those with a low risk score in TCGA databases. **E** The AUCs of the risk model for predicting 1-, 3- and 5-year survival were 0.79, 0.83 and 0.69, respectively, and **F** the prognosis of the high-risk group was worse in GSE17537 datasets. **G** The model could significantly distinguish high- and low-risk groups by T stage, M stage, N stage, stage, age and cancer status

Next, we performed Cox regression analyses among the 615 candidate genes in the red module above. The R-package survival Cox function was used to carry out a univariate Cox proportional hazards regression model, and 10 genes were obtained (*p* < 0.001) (Supplementary Table S6). Then, Lasso regression was used to solve the multicollinearity problem during regression analysis and reduce the number of genes in the risk model. We used the glmnet package to perform Lasso Cox regression analysis and the change trajectory of each independent variable (Fig. 7A). As lambda gradually increases, the number of independent variable coefficients tends to gradually increase. Next, we used a tenfold cross test to construct the model and confidence interval under each lambda (Fig. 7B). The model was optimal when lambda = 0.0175, and 8 genes were chosen to construct a risk model.

The final 8-gene model is as follows:

$$\mathrm{RiskScore}=0.225*\mathrm{PBXIP}1+0.311*\mathrm{MPZ}+0.065*\mathrm{SCARA}3-0.212*\mathrm{INA}+0.136*\mathrm{ILK}+0.158*\mathrm{MPP}2+0.024*\mathrm{L}1\mathrm{CAM}-0.004*\mathrm{FLNA}.$$

The patients were stratified into high- and low-risk groups according to the best cut-off value of the risk score in TCGA-CRC databases. To investigate the diagnostic accuracy of the prognostic risk model, the areas under the time-dependent ROC curves (AUCs) were computed. The AUCs of the risk model for predicting 1-, 3- and 5-year survival were 0.72, 0.70 and 0.65, respectively (Fig. 7C). In addition, patients with a high risk score presented significantly worse OS than those with a low risk score (Fig. 7D). To determine the accuracy and robustness of the model, we used the GSE17537 dataset as the training set. The AUCs of the risk model for predicting 1-, 3- and 5-year survival were 0.79, 0.83 and 0.69, respectively (Fig. 7E). The prognosis of the high-risk group was worse (Fig. 7F). We further performed correlation analysis of the 8-gene model among clinical factors and found that the model could significantly distinguish high- and low-risk groups by T stage, M stage, N stage, stage, age and cancer status (*p* < 0.05, Fig. 7G). These findings further show that our model has good predictive ability for some different clinical factors.

### GSVA and TMB analysis between the low- and high-risk groups

**Fig. 8:**
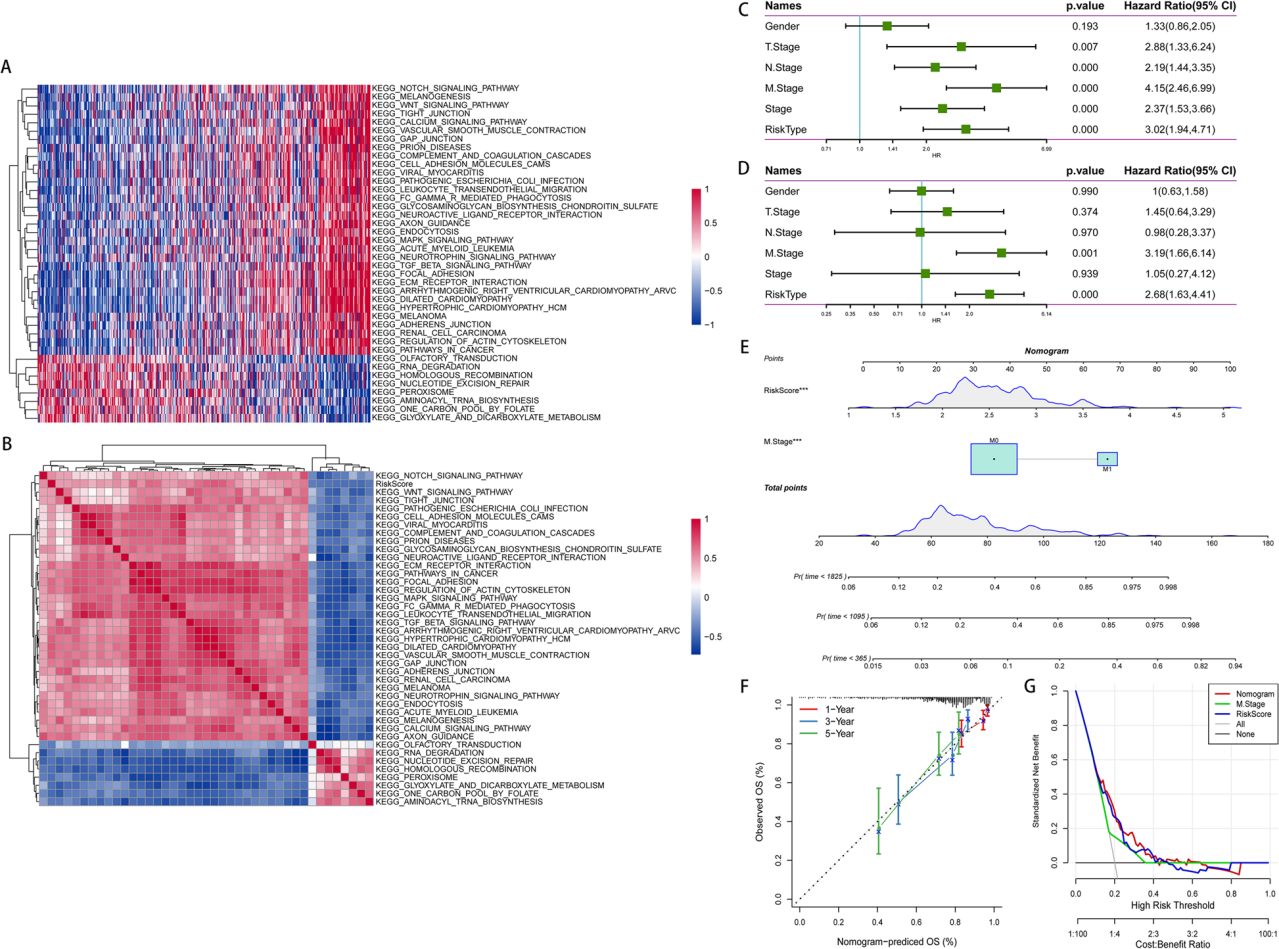
The relationship between the risk score and clinical application. **A** The correlations between biological function and functions with a correlation greater than 0.4 were selected. **B** The results showed that RENAL_CELL_CARCINOMA and other related pathways increased with the risk score. The results of **C** univariate and **D** multivariate Cox regression indicated that the risk score was an independent prognostic factor for OS. **E** The risk score features have the greatest impact on survival prediction in the nomogram model. **F** The calibration curves for the nomogram for 1-, 3- and 5-year survival were almost identical to the standard curve. **G** The results of the DCA diagram show that the nomogram has a better evaluation effect than the others

To explore the relationship between the risk score and biological function in different samples, we performed ssGSEA using the “GSVA” R package. We calculated the scores on different features and obtained the corresponding ssGSEA score of each sample (Supplementary Table S7). The correlations between biological function and risk scores were further calculated (Supplementary Table S8), and functions with correlations greater than 0.4 were selected as shown in Fig. 8A. Enrichment analysis of risk score groups revealed that 8 pathways were negatively correlated and 32 pathways were positively correlated with sample risk scores. In addition, cluster analysis of enrichment scores was carried out based on 40 KEGG pathways. The results showed that RENAL_CELL_CARCINOMA and other related pathways increased with the risk score (Fig. 8B).

We used mutect2 software to process the TCGA mutation data and calculate the tumour mutation load (TMB) of patients. There was no difference in TMB between the different molecular subtypes (Fig. S5A). In addition, we quantified the difference in the number of mutant genes (Fig. S5B), and there was no difference. Furthermore, we screened 12,489 genes with mutation frequencies greater than 3 (Supplementary Table S9) and screened genes with significant high-frequency mutations in each subtype by the chi square test, and the selection threshold was *p* < 0.05. Finally, 464 genes (Supplementary Table S10) were obtained. The mutation characteristics of the top 15 genes in each subtype are shown in Fig. S5C.

### Construction of a nomogram integrating the risk score and clinical features

To assess the independence of the 8-gene model for clinical application, we used univariate (Fig. 8C) and multivariate (Fig. 8D) Cox regression to analyse the clinical information and risk score. The results indicated that the risk score was an independent prognostic factor for OS (HR = 2.68, 95% CI = 1.63–4.41, *p* < 0.05) in the TCGA-CRC database. According to the results of univariate and multivariate analyses, we constructed a nomogram model with clinical features (M stage and risk score) (Fig. 8E). In the model, the risk score features have the greatest impact on survival prediction, which indicates that the 8-gene risk model can better predict prognosis. As shown in Fig. 8F, the calibration curves for the nomogram for 1-, 3- and 5-year survival were almost identical to the standard curve. In addition, we used decision curve analysis (DCA) to evaluate the reliability of the model. The results of the DCA diagram showed that the nomogram had a better evaluation effect than the others (Fig. 8G). Furthermore, we performed Kaplan‒Meier survival analysis according to age, male sex, female sex, T stage, N stage, M stage and stage in the TCGA-CRC databases. Patients were stratified into the following subgroups: age > 65 or <  = 65, male, female, T1-2 stage, T3-4 stage, N1-3 stage, N0 stage, M0 stage, M1 stage, MI-II stage, and MIII-IV stage. The OS of patients in the high-risk group was significantly shorter than that of patients in the low-risk group in the age > 65 or <  = 65, male, female, T3-4 stage, N1-3 stage, N0 stage, M0 stage subgroup and MI-II stage subgroups. The above findings further showed that our risk model still has good predictive ability in different clinical clusters (Fig. S6).

### Expression and function analysis of the previously unreported model genes MPZ, SCARA3, MPP2 and PBXIP1 in CRC

**Fig. 9:**
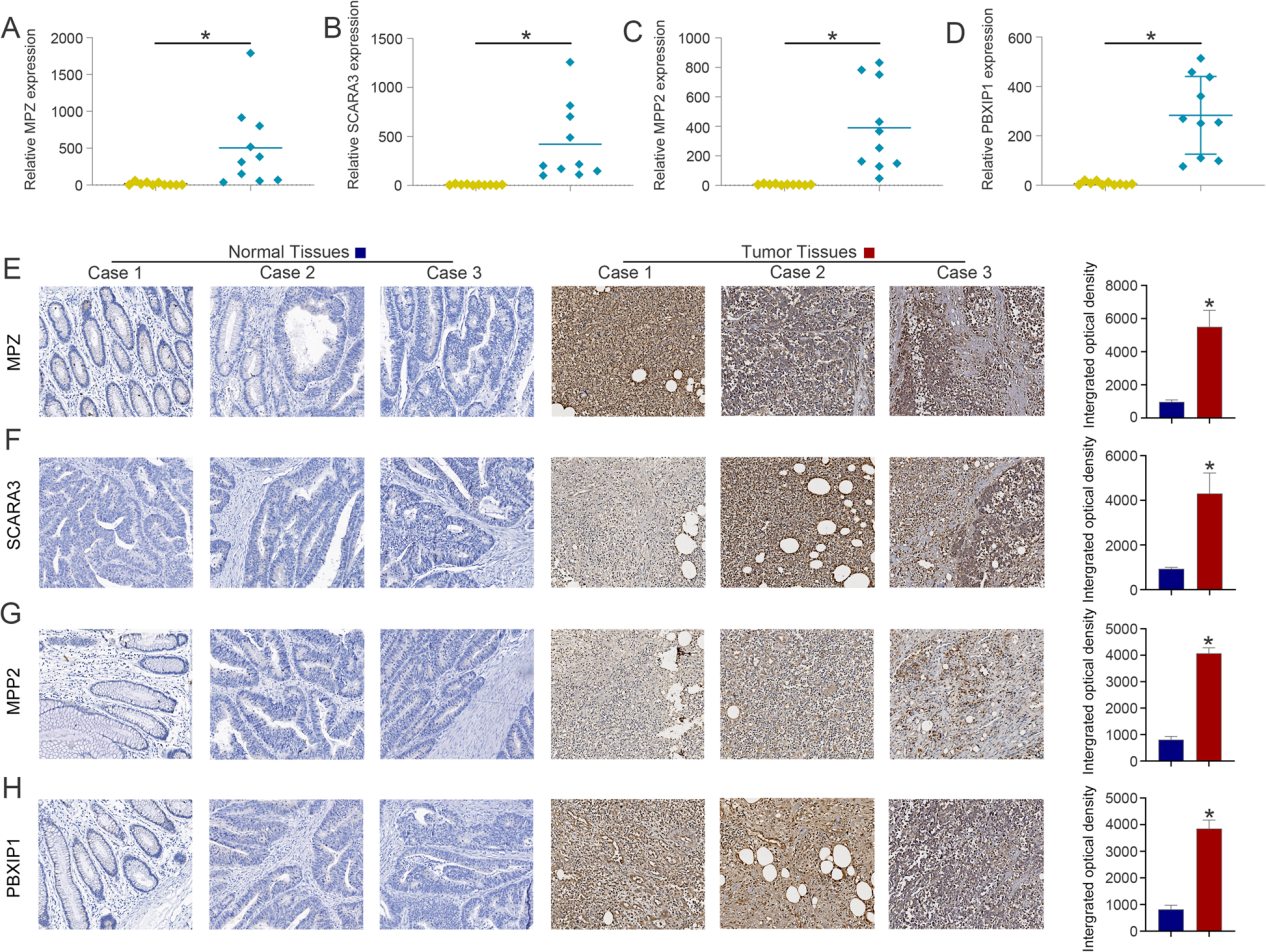
The expression of the unreported model genes. The results of **A-D** qPCR and **E–H** IHC analysis showed that the expression of MPZ, SCARA3, MPP2 and PBXIP1 was high in CRC tissues

Furthermore, we examined the expression profiles of previously unreported model genes (MPZ, SCARA3, MPP2 and PBXIP1) in clinical samples from CRC patients by qPCR (Fig. 9A-D) and IHC (Fig. 9E-H) analysis and found that the expression levels of MPZ, SCARA3, MPP2 and PBXIP1 were high in CRC tissues.

**Fig. 10:**
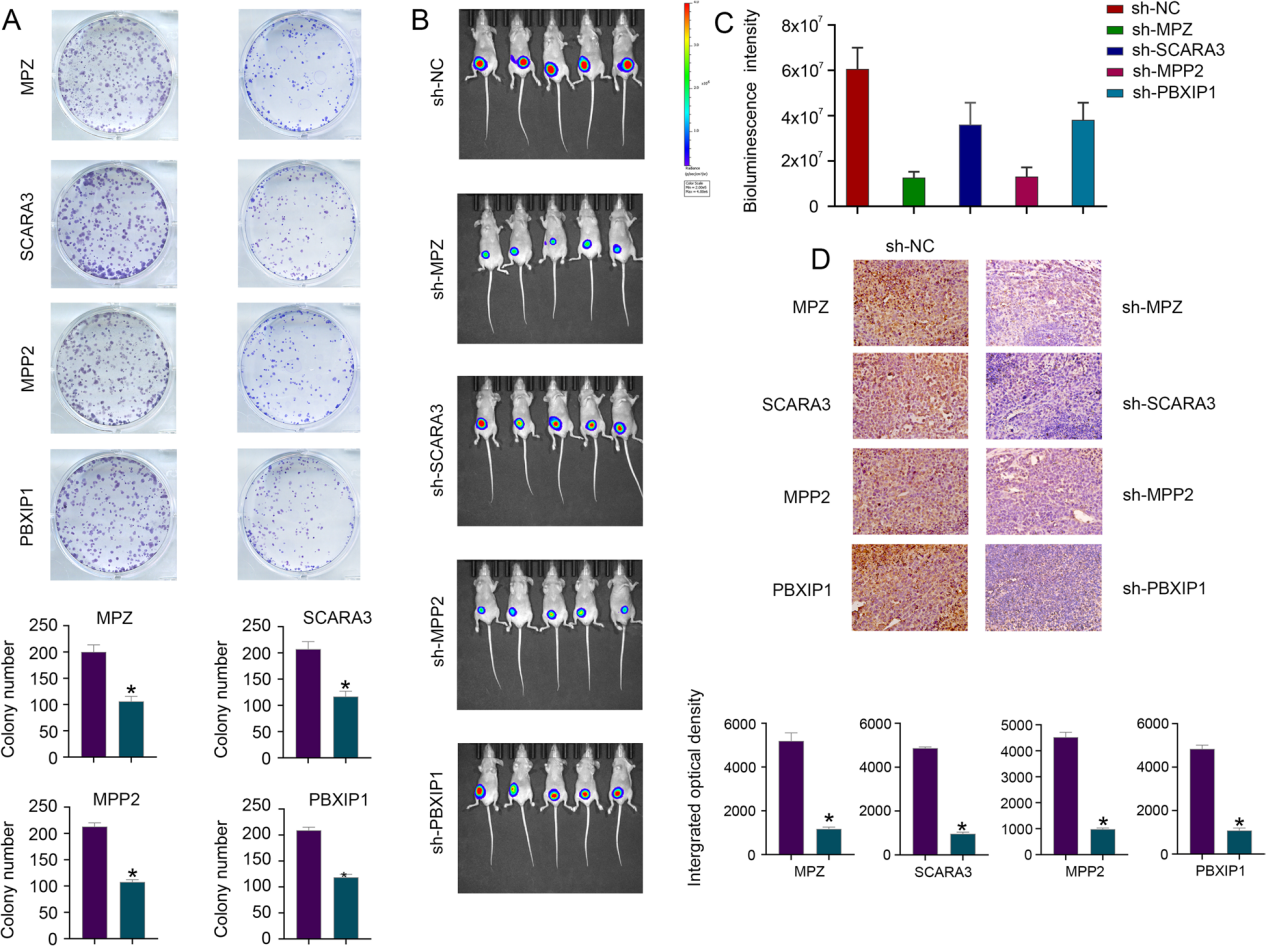
The functional role of MPZ, SCARA3, MPP2 and PBXIP1 in CRC cells. The results indicated that the inhibition of MPZ, SCARA3, MPP2 and PBXIP1 expression inhibited the colony formation ability of SW620 cells **A** in vitro and **B-C** tumorigenicity in vivo. **D** IHC analysis of xenografted tumour tissues revealed that MPZ, SCARA3, MPP2 and PBXIP1 expression was low in the SW620/sh-MPZ, SW620/sh-SCARA3, SW620/sh-MPP2 and SW620/sh-PBXIP1 groups

To clarify the functional roles of MPZ, SCARA3, MPP2 and PBXIP1 in CRC cells, a clone formation assay and orthotopic colorectal tumour model were applied to detect the regulatory roles of these previously unreported model genes. Lentiviral expression vectors (pLKO.1-NC-GFP, pLKO.1-MPZ-GFP, pLKO.1-SCARA3-GFP, pLKO.1-MPP2-GFP or pLKO.1-PBXIP1-GFP) were used to construct MPZ (sh-NC and sh-MPZ), SCARA3 (sh-NC and sh-SCARA3), MPP2 (sh-NC and sh-MPP2) or PBXIP1 (sh-NC and sh-PBXIP1) interference cell lines in SW620 cells, and SW620 cells transfected with sh-NC were considered the control. Western blots were used to validate the cell transfection efficiency and the results are presented in Fig. S7A. Lentiviral expression vectors (pLVX-IRES-Neo-3xFlag, pLVX-MPZ-IRES-Neo-3xFlag, pLVX-SCARA3-IRES-Neo-3xFlag, pLVX-MPP2-IRES-Neo-3xFlag or pLVX-PBXIP1-IRES-Neo-3xFlag) were used to over-express MPZ (vector and MPZ), SCARA3 (vector and SCARA3), MPP2 (vector and MPP2) or PBXIP1 (vector and PBXIP1) in the CRC cell line SW480, and SW480 cells transfected with vector were considered the control. The results indicated that the inhibition of MPZ, SCARA3, MPP2 and PBXIP1 expression inhibited the colony formation ability of SW620 cells in vitro (Fig. 10 A) and tumorigenicity in vivo (Fig. 10 B-C), while overexpression of MPZ, SCARA3, MPP2 and PBXIP1 promoted the colony formation ability of SW480 cells in vitro (Fig. S7B) and tumorigenicity in vivo (Fig. S7C)*.* IHC analysis of xenografted tumour tissues revealed that MPZ, SCARA3, MPP2 and PBXIP1 expression levels were low in the SW620/sh-MPZ, SW620/sh-SCARA3, SW620/sh-MPP2 and SW620/sh-PBXIP1 groups (Fig. 10D).

## Discussion

The heterogeneity of tumours plays critical roles in tumour progression and treatment response and is an opportunity for cancer diagnosis and treatment [11]. The utility of scRNA-seq helps us to better understand tumour heterogeneity and may bring promising prospects for clinical diagnosis and therapy. A growing body of studies have identified that scRNA-seq can be utilized to explore genetic alterations in CRC. Zhou Y. reported that five genes (BGN, RCN3, TAGLN, MYL9, and TPM2) were identified as fibroblast-specific biomarkers of poorer prognosis of CRC using single-cell multiomics sequencing of FACS-sorted cells isolated from CRC patients [12]. Single-cell RNA sequencing analysis showed that the intratumoral immunomodulation of CD73 inhibition is distinct from PD-1 inhibition and exhibits potential as a novel anticancer immunotherapy for CRC [13]. However, a new prognostic model was constructed by single-cell RNA sequencing, and bulk transcriptome data of CRC samples have yet to be obtained.

In this study, we developed a prognostic model for CRC patients by integrating scRNA-seq and bulk RNA-seq data. First, we collected the scRNA-seq profiles of 13 CRC samples with 43,851 cells and obtained 20 cell clusters. Meanwhile, the results of survival analysis showed that the high abundances of C4, C11 and C13 and the low abundances of C5 and C14 resulted in better survival. The WGCNA results showed that the red module was most related to the tumour and the C14 cluster, which contains 615 genes. Next, we performed Lasso Cox regression analysis among the 615 candidate genes, and an 8-gene risk model was constructed. The time-dependent ROC curves and survival analysis revealed that the 8-gene risk model can better predict prognosis in the TCGA and GSE17537 datasets. Furthermore, our risk model still has good predictive ability in different clinical clusters. Finally, we examined the expression profiles of the previously unreported model genes and showed that the expression levels of MPZ, SCARA3, MPP2 and PBXIP1 were high in CRC tissues. The functional experiment results indicated that the inhibition of MPZ, SCARA3, MPP2 and PBXIP1 expression could inhibit the colony formation ability of SW620 cells in vitro and tumorigenicity in vivo, while overexpression of MPZ, SCARA3, MPP2 and PBXIP1 promoted the colony formation ability of SW480 cells in vitro and tumorigenicity in vivo*.* These data revealed that the new prognostic model could potentially be used for clinical application and provide potential therapeutic targets for CRC patients.

Consistent with other studies, INA, ILK, L1CAM and FLNA were abnormally expressed and played regulatory roles in the progression of CRC. For example, INA is a novel tumour suppressor that increases microtubule polymerization during CRC progression [14]. ILK overexpression in human CRC is associated with EMT and CSC traits, contributing to tumour progression and chemoresistance [15]. L1CAM defines the regenerative origin of metastasis-initiating cells in colorectal cancer [16]. FLNA could be a novel and reliable CRC marker and a potential therapeutic target against CRC [17]. Our results were consistent with previous reports. For unreported model genes (MPZ, SCARA3, MPP2 and PBXIP1), recent studies have demonstrated that those genes are involved in the development of cancers. Haas GP. reported that six cores each from the MPZ were most likely to detect the majority of clinically significant cancers but also detected many insignificant cancers [18]. The upregulation of SCARA3 during disease progression from diagnosis to recurrence suggests that it plays a role in ovarian cancer biology [19]. The overexpression level of MPP2 in liver cancer cells promotes their apoptosis [20], and Huang C. reported that MPP2 is related to the 5-year survival rate of colon cancer patients [21]. A recent study revealed that PBXIP1 is a novel protein overexpressed in astrocytoma and ependymoma that is involved in tumour cell proliferation and migration and warrants further exploration as a novel therapeutic target in these tumours [22]. However, the expression profiles of MPZ, SCARA3 and PBXIP1 and the roles of MPZ, SCARA3, MPP2 and PBXIP1 in CRC remain elusive. In the present study, our results showed that the expression levels of MPZ, SCARA3, MPP2 and PBXIP1 were high in CRC tissues, and the inhibition of MPZ, SCARA3, MPP2 and PBXIP1 expression could inhibit the colony formation ability of SW620 cells in vitro and tumorigenicity in vivo, while overexpression of MPZ, SCARA3, MPP2 and PBXIP1 promoted the colony formation ability of SW480 cells in vitro and tumorigenicity in vivo*.* Consistent with our risk observations, the functions of these specifically expressed markers were primarily as potential oncogenes in CRC.

We are aware of several limitations in this study. First, the number of patients in this study was relatively small. The gene signature needs to be validated further in multicentre trials and larger patient cohorts. Second, due to technical limitations, we cannot uncover the underlying mechanism research on unreported genes. Further experiments need to be conducted to verify our analysis results in the future.

## Conclusions

In summary, by scRNA-seq and bulk RNA-seq data, and performing WGCNA, a novel prognostic model for OS prediction in CRC patients could be applied to predict the survival probability of CRC patients. Subsequently, we also explored the roles of 4 previously unreported genes (MPZ, SCARA3, MPP2 and PBXIP1), which could serve as new treatment targets for CRC in the future.

## Supplementary Information

**Additional file 1: Fig. S1.** (A-D): Quality control chart before and after filtration. (E): dim=30, and the cells were clustered by the FindNeighbors and FindClusters function (Resolution=0.4), and 20 clusters were obtained.

**Additional file 2: Fig. S2.** (A): The first three marker genes to draw a violin diagram and (B): the key pathways in 2 clusters (C5 and C19). (C): The first three marker genes to draw a violin diagram and (D): the key pathways in 3 clusters (C1, C8 and C10).

**Additional file 3: Fig. S3.** (A): The first three marker genes to draw a violin diagram and (B): the key pathways in 7 clusters (C0, C2, C3, C4, C6, C15 and C16).

**Additional file 4: Fig. S4.** The key pathways in the C12, C14 and C18 clusters.

**Additional file 5: Fig. S5.** (A-B): There was no difference in TMB or the number of mutant genes between different molecular subtypes. (C): The mutation characteristics of the top 15 genes in each subtype.

**Additional file 6: Fig. S6.** The OS of patients in the high-risk group was significantly shorter than that of patients in the low-risk group in the age>65 or< =65, male, female, T3-4 stage, N1-3 stage, N0 stage, M0 stage subgroup and MI-II stage subgroups. The above findings further showed that our risk model still has good predictive ability in different clinical clusters.

**Additional file 7: Fig. S7.** (A): Western blots were used to validate the cell transfection efficiency. Overexpression of MPZ, SCARA3, MPP2 and PBXIP1 promoted the colony formation ability of SW480 cells (B): *in vitro* and (C): tumorigenicity *in vivo.*

**Additional file 8: Supplementary Table S1.** The results of the marker genes in each subgroup.

**Additional file 9: Supplementary Table S2.** The results of the 20-cluster enrichment analysis.

**Additional file 10: Supplementary Table S3.** The 615 genes in the red module.

**Additional file 11: Supplementary Table S4.** The results of GO functional enrichment and KEGG pathway analysis.

**Additional file 12: Supplementary Table S5.** The results of the cell communication among 20 cell clusters.

**Additional file 13: Supplementary Table S6.** Ten genes were identified by univariate Cox analysis.

**Additional file 14: Supplementary Table S7.** The corresponding ssGSEA score of each sample.

**Additional file 15: Supplementary Table S8.** The correlations between biological function and risk scores were further calculated.

**Additional file 16: Supplementary Table S9.** 12489 genes with mutation frequencies greater than 3.

**Additional file 17: Supplementary Table S10.** 464 genes were obtained by the selection threshold *p* < 0.05.

## Data Availability

The datasets used or analysed during the current study are available from the corresponding author on reasonable request.
