# Supplementary figures and images for: Single-cell and WGCNA uncover a prognostic model and potential oncogenes in colorectal cancer
Source: Biol Proced Online. 2022 Sep 19;24:13. doi: 10.1186/s12575-022-00175-x (PMC9484253; doi:10.1186/s12575-022-00175-x)

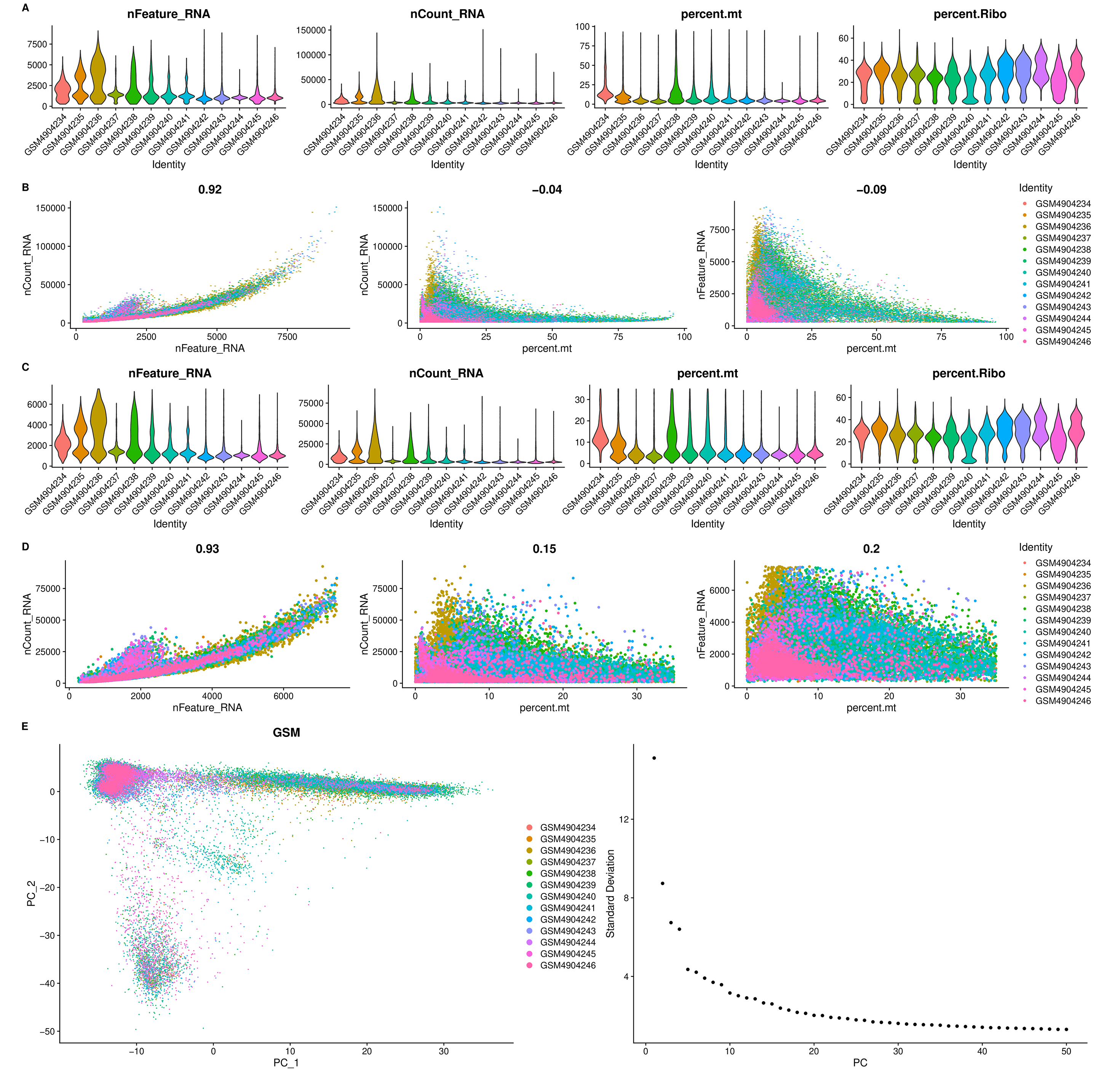

Supplement: Supplementary file 1 — Additional file 1: Fig. S1. (A-D): Quality control chart before and after filtration. (E): dim=30, and the cells were clustered by the FindNeighbors and FindClusters function (Resolution=0.4), and 20 clusters were obtained. [file 12575_2022_175_MOESM1_ESM.tif]

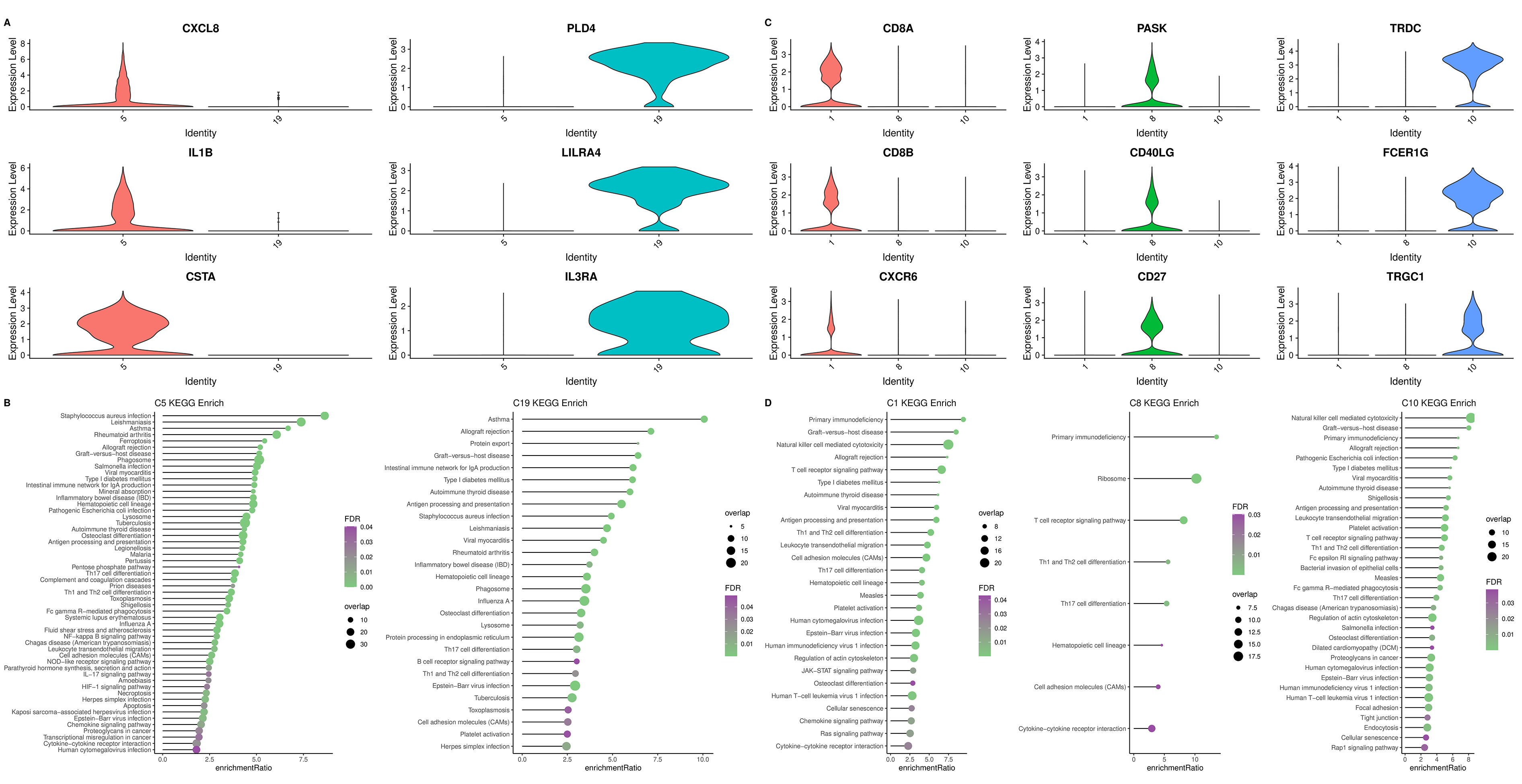

Supplement: Supplementary file 2 — Additional file 2: Fig. S2. (A): The first three marker genes to draw a violin diagram and (B): the key pathways in 2 clusters (C5 and C19). (C): The first three marker genes to draw a violin diagram and (D): the key pathways in 3 clusters (C1, C8 and C10). [file 12575_2022_175_MOESM2_ESM.tif]

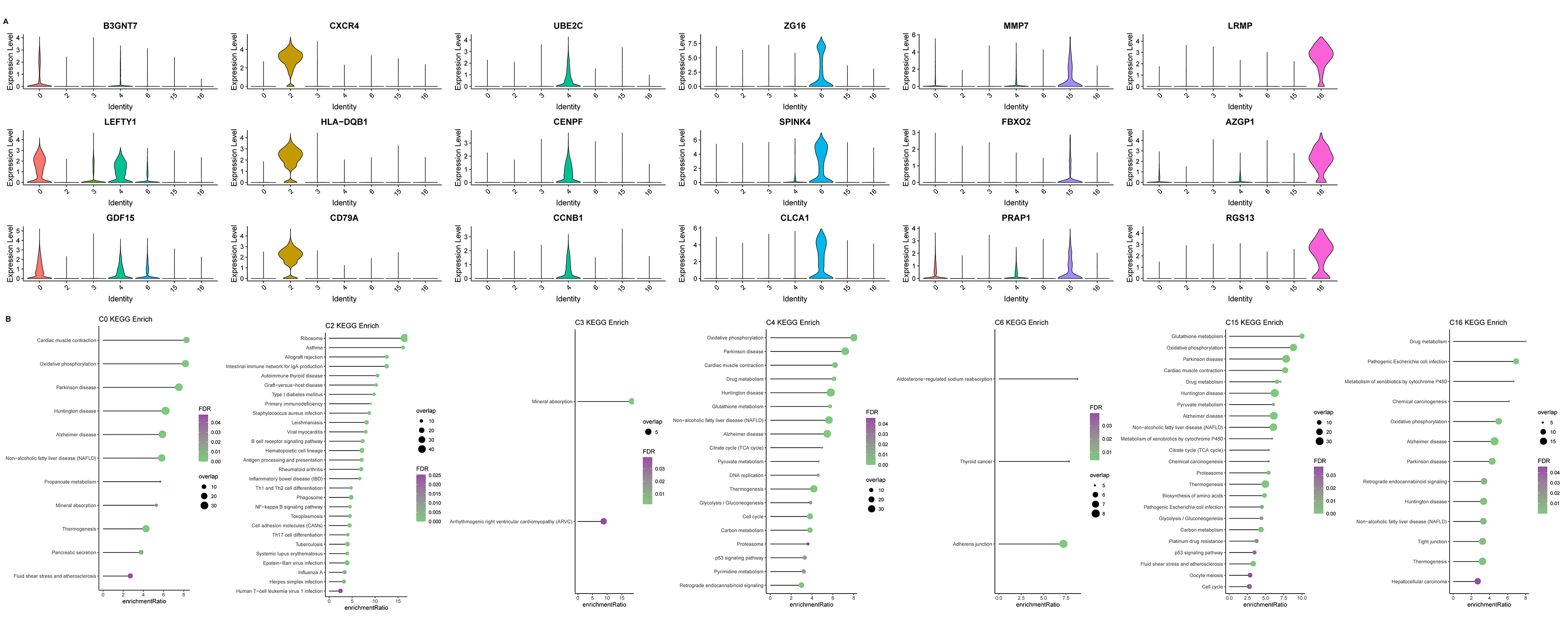

Supplement: Supplementary file 3 — Additional file 3: Fig. S3. (A): The first three marker genes to draw a violin diagram and (B): the key pathways in 7 clusters (C0, C2, C3, C4, C6, C15 and C16). [file 12575_2022_175_MOESM3_ESM.tif]

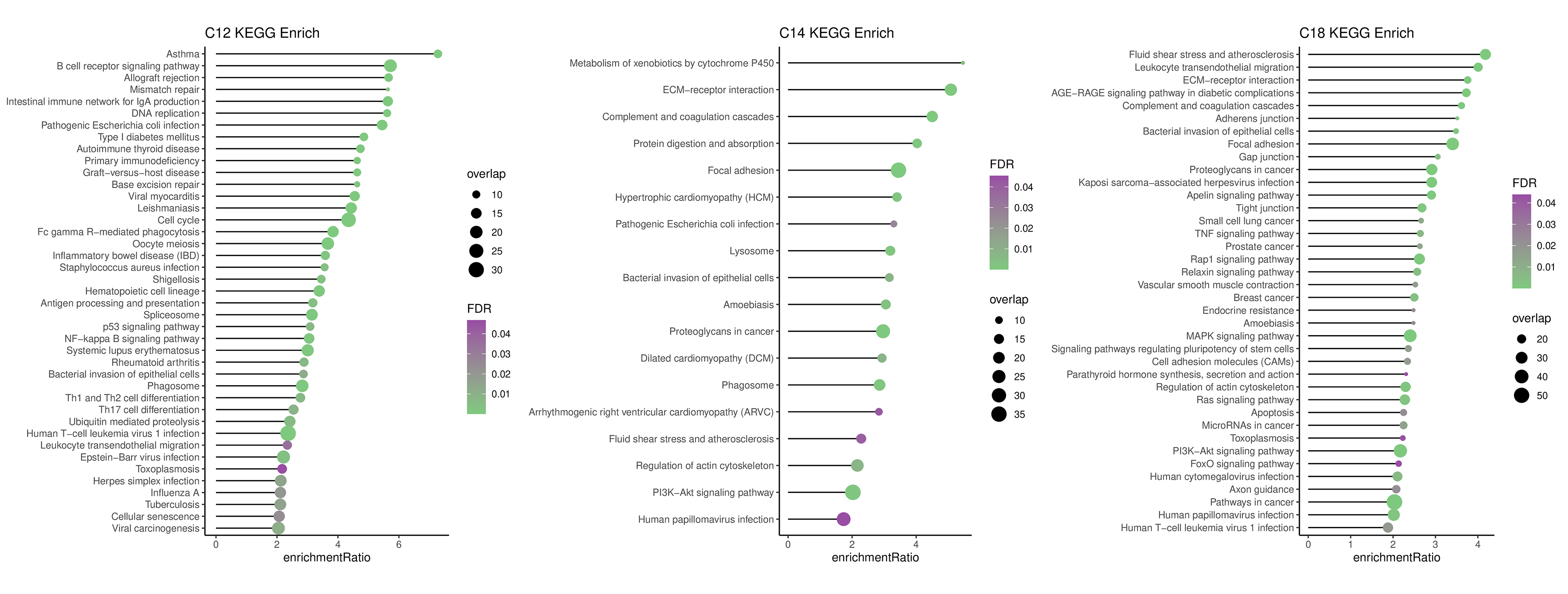

Supplement: Supplementary file 4 — Additional file 4: Fig. S4. The key pathways in the C12, C14 and C18 clusters. [file 12575_2022_175_MOESM4_ESM.tif]

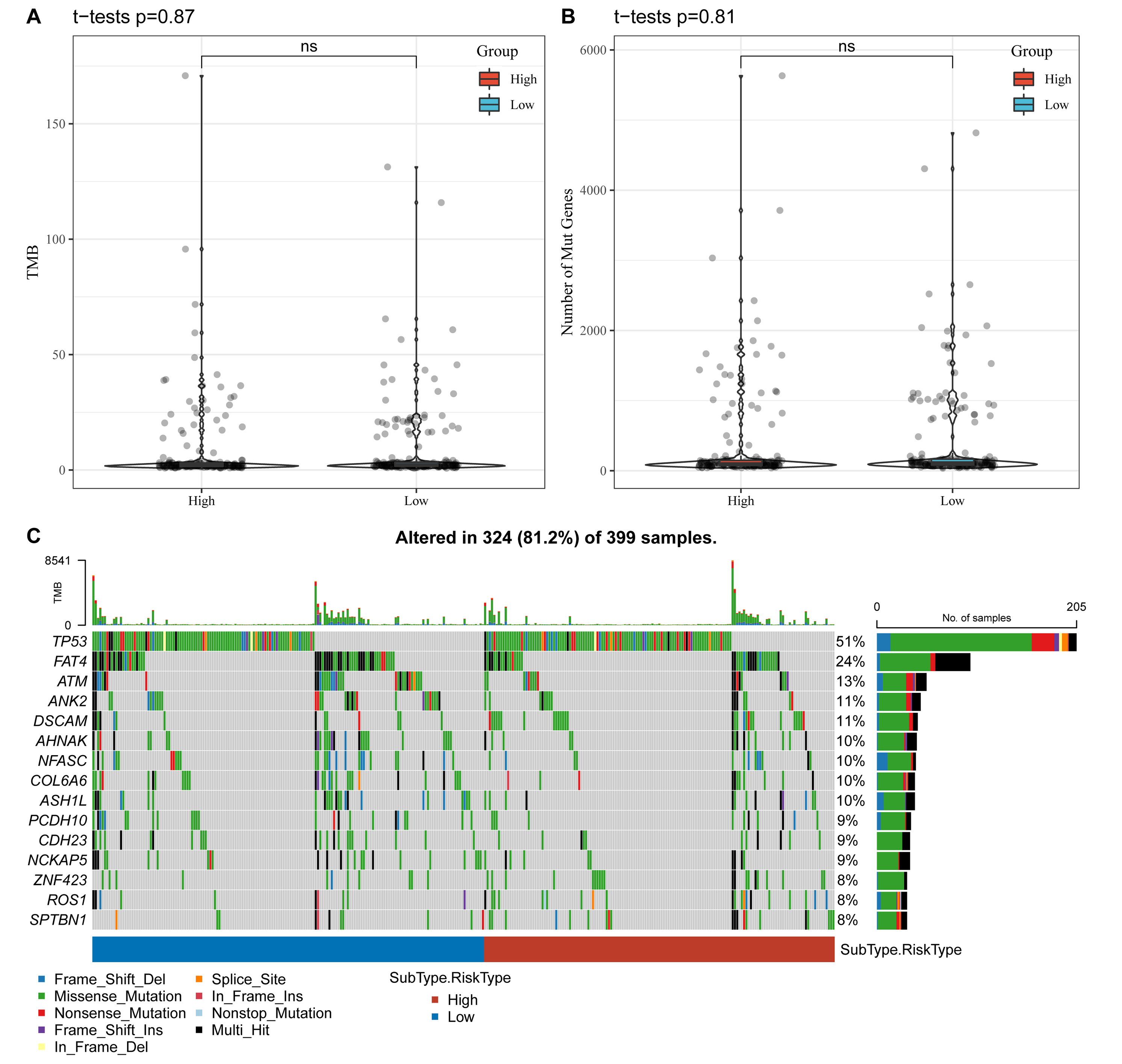

Supplement: Supplementary file 5 — Additional file 5: Fig. S5. (A-B): There was no difference in TMB or the number of mutant genes between different molecular subtypes. (C): The mutation characteristics of the top 15 genes in each subtype. [file 12575_2022_175_MOESM5_ESM.tif]

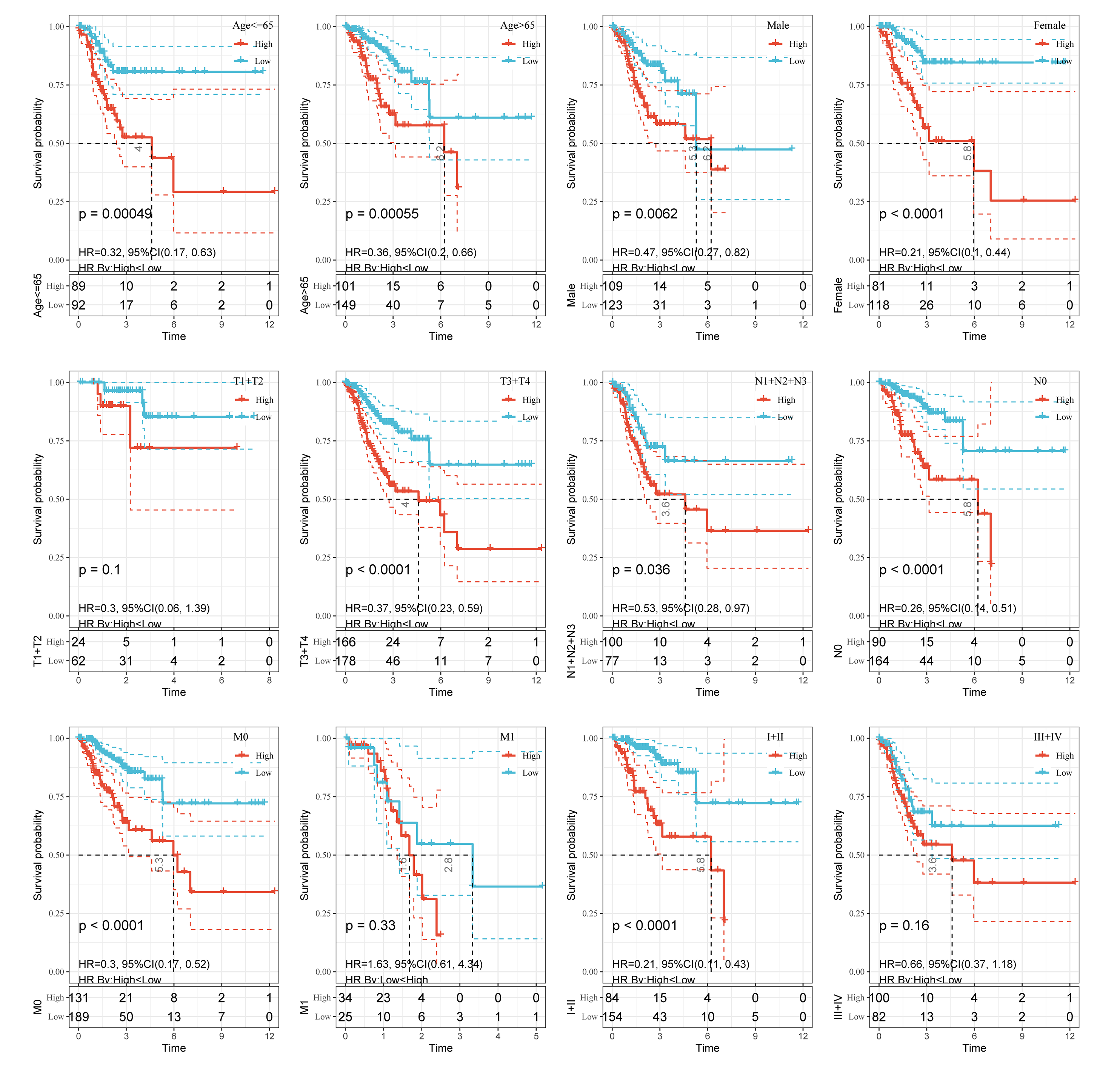

Supplement: Supplementary file 6 — Additional file 6: Fig. S6. The OS of patients in the high-risk group was significantly shorter than that of patients in the low-risk group in the age>65 or< =65, male, female, T3-4 stage, N1-3 stage, N0 stage, M0 stage subgroup and MI-II stage subgroups. The above findings further showed that our risk model still has good predictive ability in different clinical clusters. [file 12575_2022_175_MOESM6_ESM.tif]

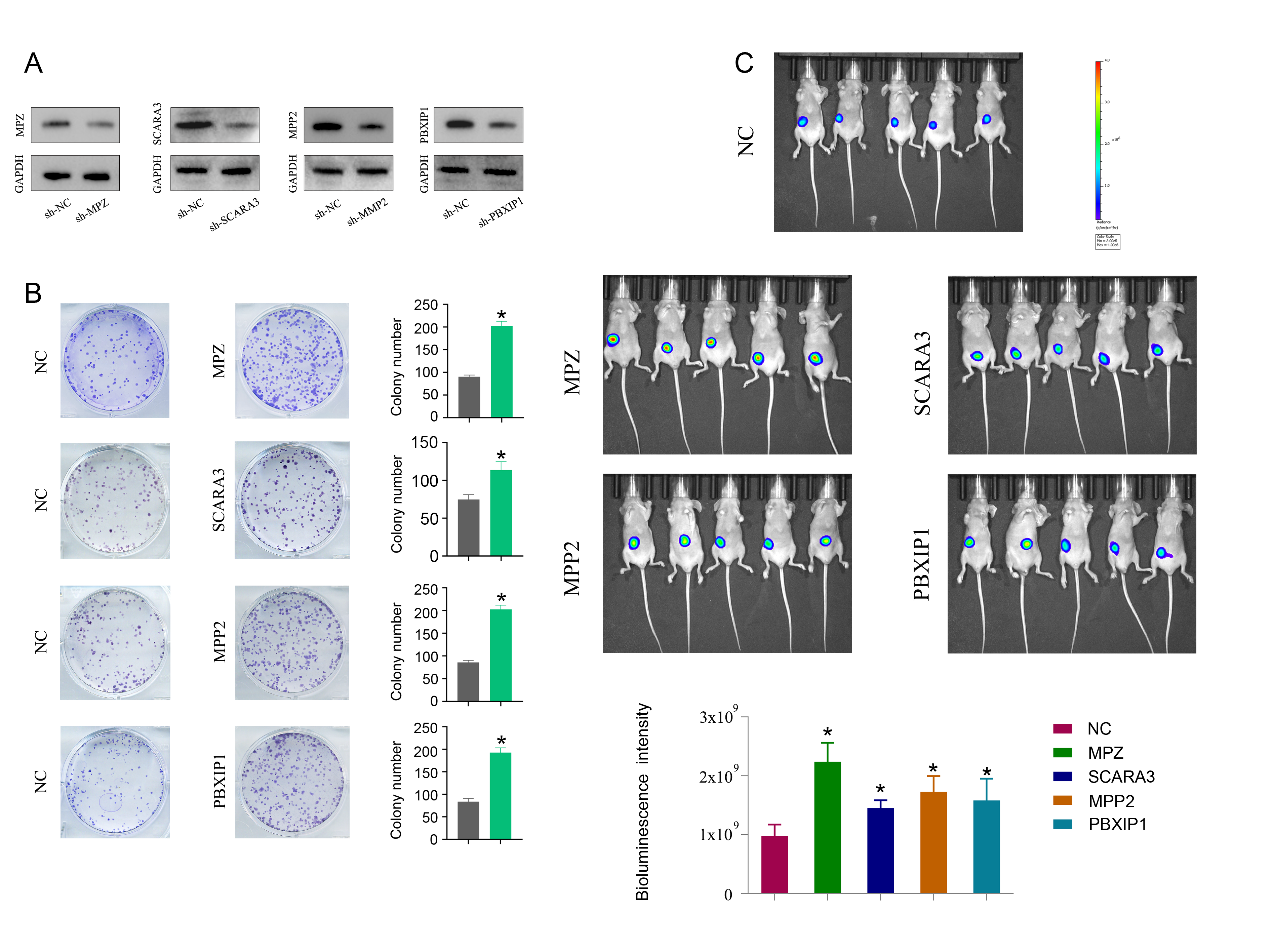

Supplement: Supplementary file 7 — Additional file 7: Fig. S7. (A): Western blots were used to validate the cell transfection efficiency. Overexpression of MPZ, SCARA3, MPP2 and PBXIP1 promoted the colony formation ability of SW480 cells (B): in vitro and (C): tumorigenicity in vivo. [file 12575_2022_175_MOESM7_ESM.tif]
